# Do objective neighbourhood characteristics relate to residents’ preferences for certain sports locations? A cross-sectional study using a discrete choice modelling approach

**DOI:** 10.1186/s12889-017-4949-5

**Published:** 2017-12-11

**Authors:** Ineke Deelen, Marijke Jansen, Nico J. Dogterom, Carlijn B. M. Kamphuis, Dick Ettema

**Affiliations:** Department of Human Geography and Spatial Planning, Faculty of Geosciences, Utrecht University, Heidelberglaan 2, Utrecht, 3584 CS The Netherlands

**Keywords:** Sports participation, Public space, Sports facility, Sports club, Environmental characteristics

## Abstract

**Background:**

The number of sports facilities, sports clubs, or city parks in a residential neighbourhood may affect the likelihood that people participate in sports and their preferences for a certain sports location. This study aimed to assess whether objective physical and socio-spatial neighbourhood characteristics relate to sports participation and preferences for sports locations.

**Methods:**

Data from Dutch adults (*N* = 1201) on sports participation, their most-used sports location, and socio-demographic characteristics were collected using an online survey. Objective land-use data and the number of sports facilities were gathered for each participant using a 2000-m buffer around their home locations, whereas socio-spatial neighbourhood characteristics (i.e., density, socio-economic status, and safety) were determined at the neighbourhood level. A discrete choice-modelling framework (multinomial probit model) was used to model the associations between neighbourhood characteristics and sports participation and location.

**Results:**

Higher proportions of green space, blue space, and the number of sports facilities were positively associated with sports participation in public space, at sports clubs, and at other sports facilities. Higher degrees of urbanization were negatively associated with sports participation at public spaces, sports clubs, and other sports facilities.

**Conclusions:**

Those with more green space, blue space or sports facilities in their residential neighbourhood were more likely to participate in sports, but these factors did not affect their preference for a certain sports location. Longitudinal study designs are necessary to assess causality: do active people choose to live in sports-facilitating neighbourhoods, or do neighbourhood characteristics affect sports participation?

## Background

In many European countries, sports clubs have a long tradition in facilitating organized sports [1]. Research has shown that club-based sports participation is associated with improved psychological and social health, above and beyond the positive effects gained from other individual forms of physical activity [2, 3]. However, societal developments, such as individualization, have had an important influence on leisure culture and lifestyle, and have led to a decline in participation in sports clubs. Hence, the importance of sports clubs as the main providers of sports activities is changing [4, 5].

These changes have led to the development of new opportunities to practice sports. Although both traditional club-based sports participation (also referred to as ‘heavy sports’) and non-club sports participation (or ‘light sports’) have increased over time, non-club sports participation has increased more rapidly [4–6]. Over the past decades, informal, unorganized, non-competitive, individual forms of sports such as running, cycling, and working out in the gym have become more popular, which has resulted in a greater variety of locations used for sports activities, including public spaces [6, 7]. According to the Eurobarometer survey 2014, the largest share of European citizens aged 15 years and older engage in sports or physical activity in informal settings, such as parks and outdoor spaces (i.e., 40%), 23% engage in health centres/gyms or other sports centres, and only 13% participate in sports or physical activity as member of a sports club [8]. In the Netherlands, data showed that in 2012, 43% of Dutch sports participants participated in sports as members of a sports club, whereas 63% (also) participated in sports individually, in non-organized settings [9]. These numbers indicate the growing importance of public spaces as facilitators of sports participation.

Increasing sports participation is a common policy goal in Western societies [10], and to stimulate participation in sports, it is important to understand which locations are used for sports participation (and by whom), as well as what determinants play a role in the use of such locations. Previous studies that investigated the determinants of sports participation have mostly investigated whether people participate in sports and how frequently they participate. These studies showed associations between sports participation and individual determinants, such as socio-demographical and psychological factors and household factors [11–13]. Additionally, characteristics of the social and physical environment (such as safety, neighbourhood socio-economic status, social network, social cohesion, access to sports facilities, urban density, and attractiveness of the physical environment) were associated with sports participation [14–16]. Other studies focused on the organization of sports as the outcome measure. For example, research has shown that women are more likely to engage in informal (light) sports in commercial or alternative settings [4, 5, 7], and adults of higher social classes and with higher incomes are more likely to engage in non-organized sports [11, 17].

Although these studies provide useful insights, we know much less about the factors that relate to the preferred locations for sports participation, such as public spaces, sports facilities (e.g., health centres/gyms) or traditional sports clubs. Of special interest are objectively measured neighbourhood characteristics, as these provide opportunities to develop environmental interventions (e.g., increase the availability of walking, jogging, and cycling trails, or improve the accessibility of sports facilities) that aim to increase sports participation. To what extent one’s residential neighbourhood is conducive to sports participation (e.g., by providing sport facilities or attractive public open spaces) may not only affect *whether* one participates in sports but also at *which location* one prefers to participate, e.g., in a city park or at a sports facility, and whether they will do so as a member of a sports club. For example, adults who live near public natural spaces with good walking trails (e.g., city parks) may be more likely to use public spaces to go for a run than adults who live near a large business park. Additionally, adults living in areas with many sports facilities and sports clubs may be more likely to practice sports in a sports club or in a private health centre/gym or swimming pool than adults living in areas with only a few available sports facilities and sports clubs.

This study aims to examine whether objective neighbourhood characteristics (i.e., proportions of land use and the availability of sports facilities in the residential neighbourhood) and objective socio-spatial neighbourhood characteristics (i.e., urban density, neighbourhood socio-economic status, and neighbourhood safety) relate to sports participation and preferred sports locations. To do so, we distinguish between 1) no sports participation, 2) sports participation in public open spaces, 3) sports participation as a member of a sports club (e.g., soccer and hockey), and 4) sports participation in public or private sports facilities (e.g., gyms and swimming pools). A discrete choice-modelling approach, which is a common approach in the literature on marketing and travel behaviour [18, 19], but relatively new in the literature on sports participation and health, is used to model associations between neighbourhood characteristics and people’s propensity to fall into one of these four different groups.

## Methods

### Participants

For this cross-sectional study, data were collected in six municipalities in the Netherlands (Amsterdam, Utrecht, Alphen aan den Rijn, Heerlen, Berkelland, and Roerdalen) in September 2014. These municipalities were selected based on their differences in size, population density, and geographical location in the Netherlands (i.e., more central vs. more peripheral) to have sufficient variation in presence, type, and accessibility of sports facilities.

Eighteen thousand adults (3000 adults per municipality), aged 18–80 years old, were randomly selected from municipal population registers. An information letter was sent to the home addresses of these adults, in which they were invited to participate in an online survey on sports participation and the use of sports locations. The online survey was used to obtain data on sports participation characteristics, principal sports location, and the personal characteristics of the respondents. Adults were asked to fill in their main type of sport, that is, the sport in which they participated most frequently during the 12 months prior to the online survey. Subsequently, they were asked where that sports activity mostly occurred (e.g., a public space, a sports club, or a registered sports facility) and if they participated as member of a sports club individually or in an informal group.

In total, 1663 respondents completed the survey (9.2% response rate). Data of respondents who met the following characteristics were excluded for analyses: participation in an inactive form of sports (e.g., bridge) (*N* = 20), participation in sports at home (*N* = 40) or at non-official sports facilities (e.g., community centres) (*N* = 64), unknown or incomplete data with regard to the postal code of their home address (*N* = 236), and other socio demographical characteristics (*N* = 69). Adults who were unable to participate in sports due to disabilities (*N* = 21) or health constraints (*N* = 12) were also excluded. Complete data were available for 1201 adults, and these respondents were included in further analyses.

### Measures

#### Sports participation at specific locations

Based on survey questions about sports participation (at least once per month vs. less than once a month), the sports location that was used most often for sports participation over the past month (i.e., a public open space, a sports club, or a sports facility) and sports club membership (yes or no), the independent variable ‘sports participation at specific locations’ was categorized in four groups: 1) no sports participation (i.e., no sports participation at all or less than once per month), 2) sports participation in public spaces, 3) sports participation as a member of a sports club, using sports club facilities, and 4) sports participation at indoor (private or public) sports facilities, without club membership, e.g., health centres/gyms and swimming pools. Sports participation in public spaces included both unorganized sports (e.g., individually, with a friend, or in a small informal group), and organized sports (e.g., in a running group but without club membership). The four different types of sports participation are further referred to as no sports participation, sports participation in public spaces, sports participation at sport clubs, and sports participation at sports facilities.

#### Objective physical and socio-spatial neighbourhood characteristics

The independent variables were objectively measured neighbourhood characteristics and included land-use data, number of sports facilities, and socio-spatial data. Land-use data of respondents’ home environments were obtained using ArcMAP 10.3.1. The coordinates of the 6-digit postal codes of respondents’ home addresses were uploaded in ArcMAP. Mean coordinates of the 6-digit postal codes (i.e., polygon features) were calculated, and Euclidean buffers of different sizes (i.e., 400, 800, 1600, and 2000 m) were drawn around these coordinates. The buffers were used to calculate the proportions of different types of land use (available from Statistics Netherlands, 2012) and the number of sports facilities (available from the Dutch Facility Monitor Sport (FMS), see [20]). The following types of land use were distinguished, as it is plausible that these may be related to sports behaviour: roads, facilities (e.g., churches, hospitals, shops, restaurants, and educational institutes), green space (e.g., parks, allotments, forest, and moorland), and blue space (e.g., rivers, lakes, and sea). These land use types were chosen based on associations shown in previous literature with physical activity [21, 22] and sports participation [23], as well as based on their potential relation with sports behaviour, for instance due to the exercise friendly design of public spaces. As there is no consensus on buffer size for assessing associations between environmental characteristics and sports participation, we assessed models with various Eucledian buffers (i.e., 400, 800, 1600, and 2000 m). Based on the following reasons we decided to use the 2000 m buffers. First, the model with the 2000-m buffer had the best model fit compared to the models we have tested with other buffer sizes (McFadden R^2^, see Table 2). Second, the 2000-m buffer size corresponded best to our assumptions. We assumed that sports participants using the public space (for sports such as running et cetera) usually go further than their immediately neighbourhood of 400 or 800 m around their homes. For instance, a previous study found that runners not only use the public space in their neighbourhood, however, they also go outside the neighbourhood and out of town [24]. Moreover, our data showed that sports participants using sports clubs or private sports facilities on average travelled 3082 m (SD = 3.843 m) to their sports activities (see also [16] based on the same data), and those who use the public space for sports such as running will probably use an even wider area around their homes.

The socio-spatial data included urban density, neighbourhood socio-economic status (SES), and safety. Urban density was estimated as the average number of addresses within a radius of one square kilometre (available from Statistics Netherlands [25]). Three categories of address density were distinguished: rural (< 500 addresses per km^2^), hardly to moderately urbanized (500–1500 addresses per km^2^), and strongly to extremely urbanized (> 1500 per km^2^). Objectively measured neighbourhood safety (on 4-digit postal code level) was obtained from the ‘Leefbaarometer 2.0’ [26]. This measure includes items such as reported demolitions, crime and theft. Neighbourhood safety (mean = −0.002, SD = 0.13) was defined relative to the Dutch average score that had a standardized score of zero and was classified into three categories: safety level below the national average (score ≤ −0.05), approximately equal to the national average (score − 0.049–0.049) and above the national average (score ≥ 0.05). Neighbourhood SES (mean = −0.043, SD = 1.20), on 4-digit postal code level, was obtained from The Netherlands Institute for Social Research [27]. The SES scores were based on an aggregated indicator consisting of the following variables derived from Statistics Netherlands: average neighbourhood income, proportion of residents with a low income, proportion of residents with a low education level, and proportion of unemployed residents. We categorized the SES scores relative to the Dutch average score into three categories: neighbourhoods with an SES below (< −-1), approximately equal to (−-0.99–0.99) and above (> 1) the national average.

#### Confounders

We controlled for the following demographical characteristics: age, gender, education, having children who live at home (yes or no), and employment (yes or no). Education was classified into three levels based on the self-reported highest level of completed education: 1) lower education (i.e., no education, primary education, and lower professional education), 2) middle education (i.e., intermediate and higher general education), and 3) higher education (i.e., higher professional education and university).

#### Statistical analysis

SPSS 23.0 was used to provide descriptive statistics on respondents’ personal characteristics and objective neighbourhood characteristics (i.e., socio-spatial characteristics and proportions of land use). The influence of the discussed determinants on the use of different locations for sports participation (i.e., participants belonging to one of the four distinguished type of sports participant categories) was analysed through the application of a discrete choice modelling approach. In this approach, type of sports participant is considered to be a choice out of four alternatives available: non-participants, public space participants, sports club participants, and other sports facility participants. In discrete choice modelling, individuals are assumed to choose the alternatives that provide the highest utility [18, 19]. The utility of alternative *j* (*j* = 1,…,*J*) for individual *n* can be represented by the following function:

1 $$ {U}_{nj}={\beta}^{\hbox{'}}{X}_{nj}+{\varepsilon}_{nj} $$

Here, is a vector of the observed characteristics (objective physical and socio-spatial neighbourhood characteristics as well as individuals’ socio-demographic characteristics), which is the deterministic part of the utility function and in the context of this study only includes individual-specific variables, and an error term , which is the stochastic component of the utility function. The probability that individual *n* will choose alternative *i* is the probability that the utility derived from alternative *i* exceeds the utility of the other alternatives, which can be represented by:

2 $$ {P}_{ni}=P\left(i|j\right)=P\left({\varepsilon}_{nj}-{\varepsilon}_{ni}<{\beta}^{\hbox{'}}{X}_{ni}-{\beta}^{\hbox{'}}{X}_{nj},\forall j\ne i\kern0.5em \right) $$

Under the assumption of independently and identically distributed (IID) error terms, the logit probabilities underlying the popular multinomial logit (MNL) model become:

3 $$ {P}_{ni}=\frac{{\mathit{\exp}}^{\beta^{\hbox{'}}{X}_{ni}}}{\sum_j{\mathit{\exp}}^{\beta^{\hbox{'}}{X}_{nj}}} $$

Central to the MNL model is the independence of irrelevant alternatives (IIA) property, which implies that the preference for an alternative is not affected by the inclusion or exclusion of other alternatives in the choice set. This property allows the use of independent standard normally distributed error terms and thus is fundamentally related to the IID assumption. However, many choice situations do not comply with IIA, as alternatives often share certain attributes that are unobserved by the researcher and therefore lead to correlations in the error terms of these alternatives. Additionally, in the case of sports location choice, such correlations can potentially be present. For example, being a ‘public space participant’ or a ‘other sports facility participant’ might be a shared preference of persons motivated to participate in individual sports but with a dislike for joining formal sports clubs. The Hausman-McFadden test [28] offers a procedure to test the IIA hypothesis for an MNL model, and applying this test, our data showed that the IIA property was violated for the estimated MNL model. In the presence of only individual-specific variables, the multinomial probit model (MNP) offers an attractive alternative model specification that can handle dependence across alternatives. The MNP model assumes that errors follow a multivariate normal distribution with mean 0 and covariance matrix ∑ [18]. The probabilities can be written as:

4 $$ {P}_{ni}=P\left(i|j\right)={\int}_{-\infty}^{\beta \ast {X}_1}\dots {\int}_{-\infty}^{\beta \ast {X}_{j-1}}f\left({\varepsilon}_{i1}^{\ast },\dots, {\varepsilon}_{ij-1}^{\ast}\right)\partial {\varepsilon}_{i1}^{\ast },\dots, \partial {\varepsilon}_{ij-1}^{\ast } $$

where f(.) is the probability density function of the multivariate normal distribution.

For the estimation of our MNP model, the software platform ‘R’, with the ‘mlogit’ package, has been used [29, 30].

## Results

Table 1 shows socio-demographic characteristics of the four groups of non-sports participants and sports participants using different locations and environmental characteristics of their residential neighbourhoods.

**Table 1:** Respondents’ characteristics and environmental factors

	Total study population	Non-sports participants	Sports participants		
			Public space	Sports club	Other sports facility
	(N = 1,201)	(*N* = 383)	(*N* = 313)	(*N* = 211)	(*N* = 294)
*Socio-demographical factors*					
Age (in years)					
*Mean (SD)*	51.3 (15.5)	52.8 (15.1)	51.9 (15.1)	47.9 (17.3)	51.1 (14.9)
Female (%)	54.3	54.8	46.6	53.1	62.6
Education (%)					
Low	15.9	24.5	11.8	9.5	13.6
Middle	37.4	41.0	31.9	37.9	38.1
High	46.7	34.5	56.2	52.6	48.3
Employed (%)	55.2	48.0	58.8	59.2	57.8
Children living at home (%)	32.2	31.6	27.8	41.7	31.0
*Neighbourhood characteristics*					
Municipality (%)					
Amsterdam	12.5	14.6	16.3	8.1	8.8
Utrecht	10.7	8.6	10.9	10.9	12.9
Alphen aan den Rijn	20.0	19.3	15.7	22.7	23.5
Heerlen	18.2	22.2	13.7	11.8	22.4
Berkelland	16.8	14.9	19.5	19.4	14.6
Roerdalen	21.8	20.4	24.0	27.0	17.7
Proportions of land use^*^ *Median % (IQR)*					
Roads	3.7 (2.6)	3.6 (2.6)	3.6 (2.6)	3.7 (2.7)	3.9 (3.0)
Green space	47.4 (49.6)	68.5 (44.1)	46.6 (57.1)	46.0 (54.3)	40.7 (54.8)
Blue space	1.9 (5.1)	0.5 (1.1)	3.6 (7.2)	3.5 (5.1)	3.9 (6.3)
Facilities	2.6 (5.8)	1.7 (4.0)	2.6 (7.6)	3.1 (6.7)	3.4 (6.9)
Sports facilities^*^ (N)	34.0 (46.0)	15.0 (33.0)	35.0 (58.0)	37.0 (56.0)	43.5 (57.0)
Urban density (%)					
Rural	47.1	44.9	49.2	54.0	42.9
Hardly – moderately urbanized	13.6	12.3	13.1	14.2	15.3
Strongly – extremely urbanized	39.3	42.8	37.7	31.8	41.8
Neighbourhood safety (%)					
Below national average (< −-0.05)	32.8	38.9	31.3	23.7	33.0
About national average (< −-0.049 – 0.049)	20.0	17.2	19.8	18.5	24.8
Above national average (> 0.05)	47.2	43.9	48.9	57.8	42.2
Neighbourhood SES status (%)					
Below national average (< −-1)	20.5	27.2	16.0	13.7	21.4
About national average (−-0.99– – 0.99)	61.2	58.0	63.6	68.7	57.5
Above national average (> 1)	18.3	14.9	20.4	17.5	21.1

Note: SD = Standard Deviation. SES = Socio-economic Status. ^*^Calculated for a 2,000 metre buffer around the centre of the 4 digit postal codes that correspond to adults’ home addresses. SES = Socio-economic Status

Table 2 shows the results of the raw (model 1) and adjusted (model 2) multinomial probit analyses. Green space, blue space, and number of sports facilities showed significant positive associations with sports participation in public space, in sports clubs, and in other sports facilities. Of these three variables, blue space showed the largest effect size. Hardly to moderate urbanity was negatively associated with sports participation in public space and in other sports facilities. Strong urbanity was negatively associated with participating in sports in public space, sports clubs, and other sports facilities. High education was positively associated with sports participation in all three types of sports locations. Significant effects of neighbourhood characteristics (i.e., regarding the direction and size) were similar for each of the sports locations.

**Table 2:** Multinomial probit model

	Model 1 – raw analyses						Model 2 – adjusted analyses					
	Public space		Sports club		Other sports facility		Public space		Sports club		Other sports facility	
*Ref = non-participants*	B	SE	B	SE	B	SE	B	SE	B	SE	B	SE
Constant	−-4.019	0.699	−-4.165	0.837	−-4.092	0.761	−-4.377	0.738	−-4.090	0.939	−-4.469	0.813
*Physical environmental characteristics* ^a^												
Roads	−-0.032	0.064	−-0.033	0.064	−-0.024	0.064	−-0.020	0.067	−-0.024	0.068	−-0.012	0.066
Facilities	0.057	0.055	0.054	0.054	0.045	0.055	0.046	0.056	0.043	0.056	0.032	0.056
Green space	**0.034** ^**^	0.006	**0.028** ^*^	0.009	**0.034** ^**^	0.006	**0.034** ^**^	0.006	**0.028** ^*^	0.010	**0.034** ^**^	0.007
Blue space	**0.650** ^**^	0.081	**0.638** ^**^	0.091	**0.652** ^**^	0.081	**0.637** ^**^	0.082	**0.625** ^**^	0.091	**0.639** ^**^	0.082
Number of sports facilities	**0.030** ^*^	0.010	**0.027** ^*^	0.011	**0.032** ^*^	0.011	**0.031** ^*^	0.011	**0.028** ^*^	0.012	**0.033** ^*^	0.011
*Socio environmental characteristics* ^b^												
Urbanity (ref. = rural)												
Hardly – moderately urbanized	−-0.677	0.359	−-0.598	0.379	−-0.681	0.356	**−-0.740** ^*^	0.359	−-0.654	0.379	**−-0.731** ^*^	0.355
Strongly - extremely urbanized	**−-0.779** ^*^	0.357	**−-0.758** ^*^	0.371	**−-0.759** ^*^	0.356	**−-0.825** ^*^	0.360	**−-0.825** ^*^	0.374	**−-0.800** ^*^	0.359
Neighbourhood safety (ref. = below nat. average)												
About national average	−-0.117	0.504	−-0.039	0.508	−-0.084	0.497	−-0.110	0.517	−-0.004	0.526	−-0.078	0.509
Above national average	0.607	0.542	0.840	0.543	0.658	0.534	0.601	0.550	0.872	0.556	0.647	0.542
Neighbourhood SES (ref. = below nat. average)												
About national average	0.131	0.501	0.147	0.501	0.078	0.496	0.051	0.504	0.046	0.510	−-0.008	0.499
Above national average	0.430	0.519	0.363	0.522	0.385	0.509	0.304	0.534	0.199	0.538	0.260	0.524
*Socio-demographic factors*												
Age (in years)							0.002	0.005	−-0.007	0.006	0.001	0.005
Female							−-0.098	0.132	−-0.098	0.127	−-0.007	0.145
Children living at home							−-0.018	0.143	0.202	0.166	0.021	0.139
Education (ref. = low)												
Middle							0.251	0.156	0.309	0.179	0.277	0.157
High							**0.478** ^*^	0.163	**0.514** ^*^	0.194	**0.463** ^*^	0.166
Employed							0.119	0.128	−-0.009	0.143	0.103	0.126
*Error structures*												
Public Space – Sports club	0.647	0.560					0.617	0.596				
Public Space – Other sports facility	**1.005** ^**^	0.066					**1.000** ^*^	0.082				
Sports club – Sports club	0.819	0.575					0.852	0.543				
Sports club – Other sports facility	0.139	0.296					0.139	0.293				
Other sports facility – Other sports facility	0.189	0.353					0.212	0.381				
*Model fit*												
Log-likelihood	−-1276.7						−-1249.9					
McFadden R^2^	0.22117						0.23752					
Chisquare	725.12						778.73					

^a^Within a 2,000 metre buffer around the home. ^b^On postcode 4 digit level. ^*^Significance < 0.05, ^**^Significance < 0.001. The reference category was non-sports participants in all models, i.e. the categories public space, sports club, and other sports facility are all compared to non-participants. SES = Socio-economic Status

The significant correlation between the error terms of public space and other facilities shows that the multinomial probit model is an appropriate model to use to analyse the data. The correlation between public space and other sports facility participants indicates a shared preference for sports participants in public space and sports participants in other sports facilities based on non-measured factors, such as attitudes towards club membership. A potential explanation is that those who do not want to participate in organized sports because of obligations and fixed times prefer more flexible forms of sports, which may occur either in public spaces or other facilities such as gyms and swimming pools.

## Discussion

This study adds to the existing literature on relationships between neighbourhood environments and sports participation by examining whether objectively measured residential neighbourhood characteristics (e.g., availability of sports facilities, green spaces) not only relates to sports participation but also to the preferred sports location of sports participants (e.g., an indoor sports facility or a city park). The results indicate that those with more green space, blue space or sports facilities in their residential neighbourhood were more likely to participate in sports, but it did not affect their preference for a certain sports location. Neighbourhood blue space in particular was associated equally strongly with sports participation at public spaces, sports clubs, and other sports facilities.

That natural environmental characteristics (i.e., green and blue space) are related to sports participation is in line with previous research that showed that attractive and liveable environments stimulate and invite people to exercise or participate in sports [31, 32]. Moreover, such environments are associated with less experienced constraints to participating in sports more frequently [16]. The strong effect of green, and particularly blue space, on sports participation in all types of locations might be related to different types of sports that different natural areas are suitable for. For instance, previous research has shown that different green and blue areas (with different sizes) are related to different modalities and intensities of physical activity [21]. However, it would be of great interest to explore the importance of blue space for sports participation in more detail, as this may provide policy makers and urban designers with more accurate information on how to design both physical activity- and sports-friendly environments..

Moreover, higher proportions of green and blue space in the residential neighbourhood were also associated with a higher chance of sports participation in sports clubs and other public or private sports facilities. Additionally, sports facilities within a 2,000-metrem buffer around the home were positively associated with sports participation at sports facilities as well as with increased sports participation in public open spaces. Somehow, a sports-promoting neighbourhood environment increases the likelihood that residents participate in sports, but it does not affect their preference for a certain location. One explanation could be processes of ‘modelling’ and perceived social norms [33, 34]. In a sports-facilitating neighbourhood, more people are out on the streets running, biking, walking, or travelling in their active wear to their sports. Viewing these active people may make residents more likely to participate in sports themselves (‘modelling’) or make them feel that being active is the social norm, which in turn increases their likelihood to become active. Another explanation may be that people with active lifestyles choose to live in (relatively ‘blue’ or ‘green’) neighbourhoods that facilitate such lifestyles; they may not necessarily use their neighbourhood facilities to practice sports but may use facilities in other parts of the city. Due to the cross-sectional design of this study, it remains unclear whether more green or blue space and sports facilities within the residential environment contribute to an increase in sports participation at public spaces, sports clubs, and other sports facilities or if adults who participate in sports choose to live in an environment that has a certain amount of sports facilities and green or blue space available. Hence, to better inform policy and intervention development, longitudinal or retrospective research is needed.

Regarding socio-spatial neighbourhood characteristics, we only found an effect of urban density. With higher urbanity levels, the chance of participating in sports declines in all types of sports locations. We found a similar effect for hardly to moderate urbanity levels, which was negatively associated with participation in public space and other (private or public) sports facilities. In other words, the higher the urbanity degree, the more likely it is that adults do not participate in sports. This is supported by existing evidence that sports participation rates are higher in rural areas in the Netherlands [15]. Literature has explained such urbanity differences in sports participation by determinants related to socio-spatial neighbourhood characteristics, such as neighbourhood SES and safety [15, 35]. However, these associations between safety, neighbourhood SES, and sports participation were not found in the current study. This inconsistency with previous research regarding the role of safety may be due to differences in measures of safety (i.e., objective vs. subjective measures). For example, it may be that perception of safety (which is mostly how safety was measured in previous studies) plays a different role in sports participation than objectively measured safety (which is how safety was measured in this study). Although neighbourhood SES was not associated with sports participation in any of the three types of sports locations, education on the individual level was associated with sports participation in all types of sports locations. This finding is in accordance with many other studies [11, 36] and implies that the effects of green space, blue space, and sports facilities occur irrespective of education.

Strengths of this study include the novel approach within sports participation literature, focusing on an outcome variable that allows for a distinction between non-participants and different types of sports participants related to their sports location. Moreover, we used a strong analytical discrete choice approach and took various objectively measured environmental measures into account. We have checked for correlations between the independent variables and found no issues with collinearity.

The low response rate (9.2%) is a limitation of this study. The response rate was lowest in the largest municipalities of Amsterdam and Utrecht (Table 1), which is probably related to underrepresentation of adults of non-native Dutch origin in the sample (10.8% compared to 21.4% nationally in 2014) [37], who more frequently live in the larger cities. Furthermore, lower educated respondents were underrepresented as they represented 15.9% of our sample whereas their share of the total population is 33%, according to 2014 data. [25]. It is unclear how these issues affected the representativeness of the results. However, the high share of higher-educated and native Dutch adults did not lead to an overrepresentation of sports participants in this study, as according to the national Dutch figures for sports participation, 30% of adults do not participate at all or participate less than once per month in sports [9], which is similar to the percentage of non-participants in the current study. This suggests that a selection bias toward more sports minded respondents is unlikely. In addition, the fact that we correct for education level in our multivariate analysis implies that the effects of e.g. environmental factors represent the general effect across education levels in a reliable way. Another limitation of this study was that we were unable to include various buffers to compare with the 2,000-metrem buffer, due to data loss when using smaller buffers.

Future research should consider taking the use of multiple sports locations into account for adults who participate in more than one type of sport because this might have an impact on sports location choice. For instance, adults who participate in multiple sports may join a sports training programme at a sports club but may also engage in sports in a public space. With regard to buffer sizes used to calculate the proportions of land use available around one’s home, future research could investigate if different buffer sizes can be used for different land use variables to better reflect the characteristics of these land-uses for sports participation behaviour. For example, it could be that the presence of (larger) green and blue environments lead to effects on sports participation that manifest themselves at a larger distance than the presence of roads and public facilities. In addition, to gain more insight into the importance of public space for sports participation, research should investigate which, how frequently, by whom and for what reasons specific locations in public space are used for sports participation. Moreover, more insight into the motivations of participants might add to the understanding of the use of different locations for sports. Longitudinal or retrospective research is needed to gain insight into the causality of the relationships between environmental characteristics (including the difference between green and blue spaces) and sports participation (in different locations).

## Conclusions

More and more, sports participants find their way to alternatives to traditional sports clubs. This study is among the first to investigate the extent to which characteristics of the physical and socio-spatial environment are associated with the use of different locations for sports participation and whether these are traditional sports clubs, private or public sports facilities (such as gyms and swimming pools) or the more informal, flexible public spaces. We found that neighbourhood characteristics were similarly associated (i.e., their direction and size of the effect) with sports participation in public space, sports clubs and sports facilities. The more green space, sports facilities, and (especially) blue space available around the home environment, the greater the chance one participates in sports in each of the three types of sports locations. In other words, physical neighbourhood characteristics do not affect one’s preferences for a certain sports location to use most often. Furthermore, higher urbanity levels were associated with lower chances of participating in sports in all three types of sports locations. For hardly to moderate urban areas this applies especially to lower probabilities participating in sports in public space and other (private or public) sports facilities. The associations we found indicate the possibly important role that the environment may have in facilitating and stimulating sports participation. For policy makers in the sports and health domains, this underlines the importance of facilitating and investing in exercise-friendly public spaces, whether participants will use these for sports activities, or join a (municipal subsidized) sports club or other sports facility. However, longitudinal study designs are necessary to assess the causality of these associations in order to adequately inform policy makers and urban designers for intervention development.
